# Chlorpromazine-induced lupus anticoagulant unveiled by transient ischemic attack: a case report with 3-year follow-up

**DOI:** 10.1192/j.eurpsy.2026.10530

**Published:** 2026-07-31

**Authors:** X. Fan, X. Jin, T. Lyu

**Affiliations:** 1 Department of Clinical Psychology; 2 Department of Geriatrics, The Sixth Affiliated Hospital of Kunming Medical University, Yuxi, China

## Abstract

**Introduction:**

Lupus anticoagulant (LA) is an acquired, phospholipid-dependent coagulation inhibitor that prolongs the activated partial thromboplastin time (APTT) *in vitro* and increases thrombotic risk *in vivo.* First-generation antipsychotics such as chlorpromazine have been sporadically linked to LA development; nevertheless, drug-induced LA is seldom considered when APTT is incidentally prolonged, especially in older patients with neuropsychiatric comorbidities.

**Objectives:**

To report a case of persistent LA-mediated thrombotic risk revealed by a transient ischemic attack (TIA) that was ultimately attributed to covert chlorpromazine administration, and to highlight the clinical implications of antipsychotic-related coagulopathy in primary stroke prevention.

**Methods:**

Detailed case description supplemented by a non-systematic literature review on chlorpromazine-associated LA and cerebrovascular risk.

**Results:**

A 69-year-old man was admitted to the geriatrics unit with acute dizziness and gait instability, which resolved within 24 h; the working diagnosis was first-ever transient ischemic attack (TIA). Apart from long-term smoking he lacked traditional vascular risk factors (no hypertension, diabetes, atrial fibrillation, or dyslipidemia). Routine coagulation screening then revealed an isolated, markedly prolonged APTT (80.3 s) that failed to correct in a 1:1 mixing study; lupus anticoagulant was positive (standardized ratio 1.91) whereas anticardiolipin and anti-β₂-glycoprotein-I antibodies were negative. On further questioning family disclosed 30-year covert chlorpromazine 75 mg (presented as “health supplement”) stopped 40 days earlier. Considering chlorpromazine’s pro-thrombotic potential and the need to continue covert administration, antipsychotic therapy was switched to risperidone oral solution 1 mg bid dissolved in drinks and antithrombotic therapy was initiated. Over 36 months APTT fell to 72.3 s, LA ratio to 1.32; no further TIA/stroke; psychosis remained stable. Given the absence of conventional risk factors, chlorpromazine-induced LA is considered the major contributor to the index thrombotic event.

**Conclusions:**

Chlorpromazine can elicit persistent LA and thrombo-embolism even in patients without classical vascular risk factors. When APTT is unexplained, a covert drug history should be actively sought. Antipsychotic-related coagulopathy represents a reversible mechanism of cerebrovascular disease; early recognition may guide antipsychotic selection and improve stroke prevention. We propose the “APTT-LA-DRUG” checklist (Ask family, Pharmacy review, Test mixing, Look for LA, Re-evaluate Unlisted Generic drugs) to remind clinicians to evaluate psychotropic agents whenever clotting times are prolonged. Future comparative studies are needed to quantify and contrast the risks of LA induction and thrombotic events across different antipsychotic compounds.

**Disclosure of Interest:**

None Declared

